# Case Report: Hyperplastic cervical polyp with lipomatous differentiation in a dog

**DOI:** 10.3389/fvets.2025.1658919

**Published:** 2025-10-22

**Authors:** Sunhye Song, Seung-Hyun Lee, Sungsoo Kim

**Affiliations:** VIP Animal Medical Center, Seoul, Republic of Korea

**Keywords:** canine, cervical polyp, lipomatous differentiation, fatty mass, computed tomography

## Abstract

Uterine lesions containing adipose tissue are extremely rare in dogs, and cervical polyps are rarely reported in veterinary literature. This case report describes an 11-year-old intact female mixed-breed dog presenting with chronic vaginal discharge. Diagnostic imaging revealed a well-defined fat-attenuating mass in the cervix. The lesion appeared as a homogeneously hyperechoic intrauterine mass on ultrasonography and exhibited hypoattenuation with enhanced internal septa on computed tomography. Histopathological examination of specimens collected during ovariohysterectomy confirmed the presence of a hyperplastic polyp with prominent lipomatous differentiation arising from the cervix. The patient’s marked obesity and hypertriglyceridemia suggested a possible role of metabolic imbalance in the lesion’s development. To the best of our knowledge, this is the first veterinary report of canine lipomatous cervical polyps. This case expands the limited literature on adipose-containing uterine lesions in dogs and highlights the diagnostic value of multimodal imaging for their identification and characterization.

## 1 Introduction

Polypoid lesions of the female reproductive tract are uncommon in dogs and are typically associated with the endometrium or vaginal mucosa (1–8). This limited documentation may partly reflect the routine practice of ovariohysterectomy for disease prevention and behavioral management in female dogs (9). In contrast to human gynecology—where endometrial and cervical polyps are frequently encountered and often detected incidentally during routine examinations (10–12, 38)—veterinary reports are rare; in dogs (1–8), polyps are usually recognized when clinical signs such as vaginal discharge or pyometra prompt investigation, as in the present case. Consequently, the true prevalence of asymptomatic reproductive polyps in dogs may be underestimated. Similarly, uterine lesions containing mature adipose tissue have only been sporadically described in canines, and their imaging and pathological features remain poorly characterized (13–18).

Given these diagnostic limitations, reproductive tract masses in dogs may be either benign or malignant with overlapping imaging features, making accurate diagnosis and long-term monitoring essential.

This report describes a unique case of a hyperplastic polyp with prominent adipose differentiation arising from the cervix in a dog. To our knowledge, this is the first veterinary report of a lipomatous cervical polyp. This case highlights distinct imaging features, including fat-specific findings observed on radiography, ultrasonography, and computed tomography (CT), and offers insight into the possible pathogenesis of such lesions in veterinary practices, drawing parallels with mechanisms proposed in human medicine.

## 2 Case description

An 11-year-old intact female mixed-breed dog weighing 17 kg presented with a 2-month history of vaginal discharge. Physical examination was unremarkable aside from moderate fever (39.8 °C) and marked obesity (body condition score 10/10). Vaginal speculum examination revealed a small amount of mucoid vulvar discharge. Hematological and biochemical profiles were largely within normal limits, except for marked hypertriglyceridemia (410 mg/dL; reference range, 10–100 mg/dL) and mild elevations in alkaline phosphatase (547 U/L; reference range, 23–212 U/L) and alanine aminotransferase (141 U/L; reference range, 10–125 U/L) levels.

**Figure 1:**
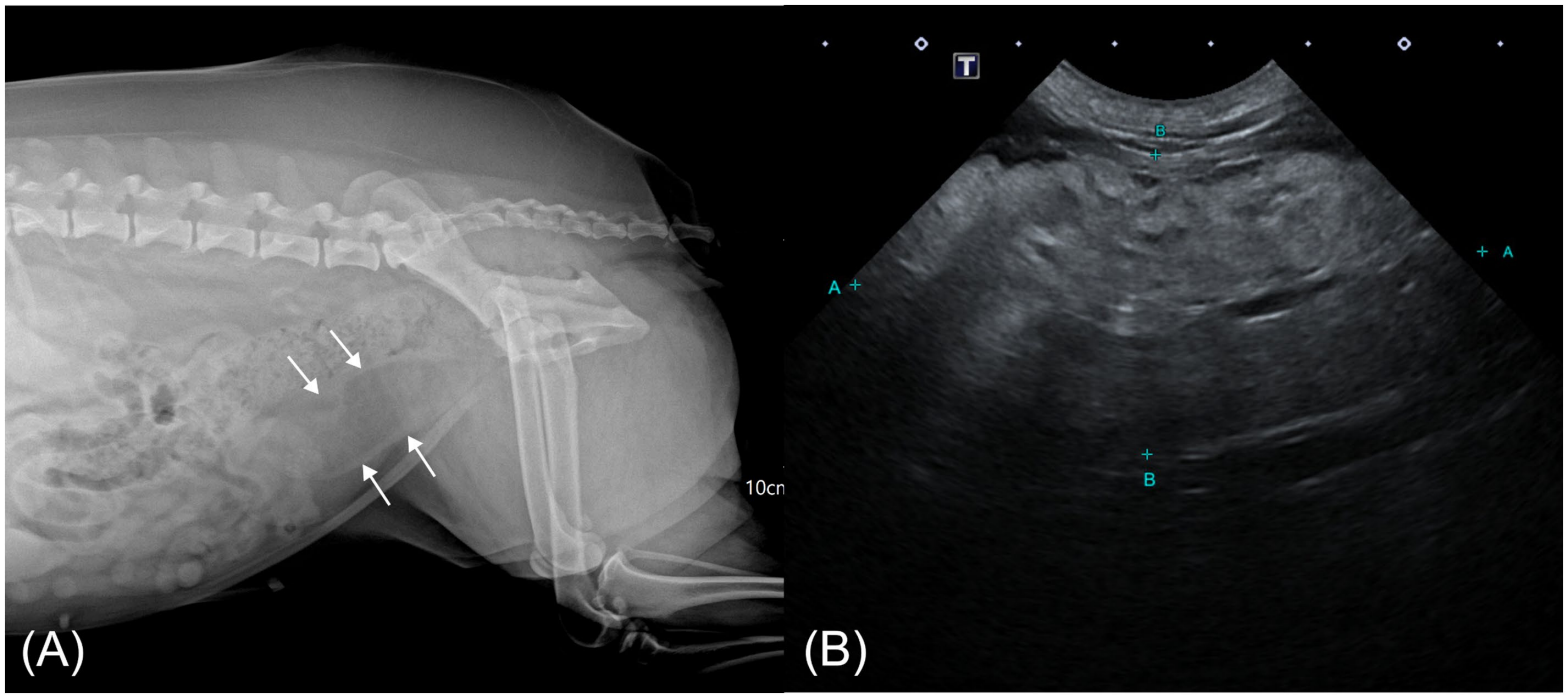
Abdominal radiographic and ultrasonographic image of a case of hyperplastic cervical polyp with lipomatous differentiation in a dog. **(A)** Right lateral abdominal radiograph showing a radiolucent tubular mass with soft tissue margins ventral to the colon (white arrow). **(B)** Transabdominal ultrasonographic image showing an oblong, predominantly hyperechoic mass cranial to the vagina with scattered linear hypoechoic foci.

Right lateral abdominal radiography revealed a tubular structure with soft tissue opacity at the margins and radiolucent content located ventral to the descending colon, extending cranially from the pelvic cavity and partially superimposed over the urinary bladder (Figure 1A). Based on its location and the clinical context, a uterine abnormality was suspected, prompting transabdominal ultrasonography (Aplio i700; Canon Medical Systems, Japan). The ultrasound showed normal ovaries and mild thickening of both uterine horns. A small volume of fluid was present within the cervical lumen. Cranial to this region, an oblong mass, presumed to be the uterine body, was identified. The mass was predominantly homogeneously hyperechogenic with scattered, short, linear hypoechoic foci and exhibited minimal vascularity on color Doppler imaging. The connection between the mass and the ventral uterine wall was indistinct (Figure 1B).

To further evaluate the exact anatomical location and characteristics of the mass, CT (Brivo CT385, GE Hangwei Medical Systems, Beijing, China; 120 kVp, 100 mA, 1.25 mm slice thickness) was performed under general anesthesia. Anesthesia was induced using butorphanol (0.2 mg/kg; Butopan Inj. 2 mg/mL; Myungmoon Pharm Co., Ltd., Seoul, Korea) and propofol (6 mg/kg; Freefol-MCT Inj. 200 mg/20 mL; Daewon Pharm Co., Ltd., Seoul, Korea), and maintained with 2% isoflurane (Isosol Inhalation Solution; Kyungbo Pharm Co., Ltd., Seoul, Korea). Pre- and post-contrast CT imaging were performed using iohexol (2.5 mL/kg; Omnipaque 300 mg I/ml; GE Healthcare Co., Ltd., China) as the contrast agent.

**Figure 2:**
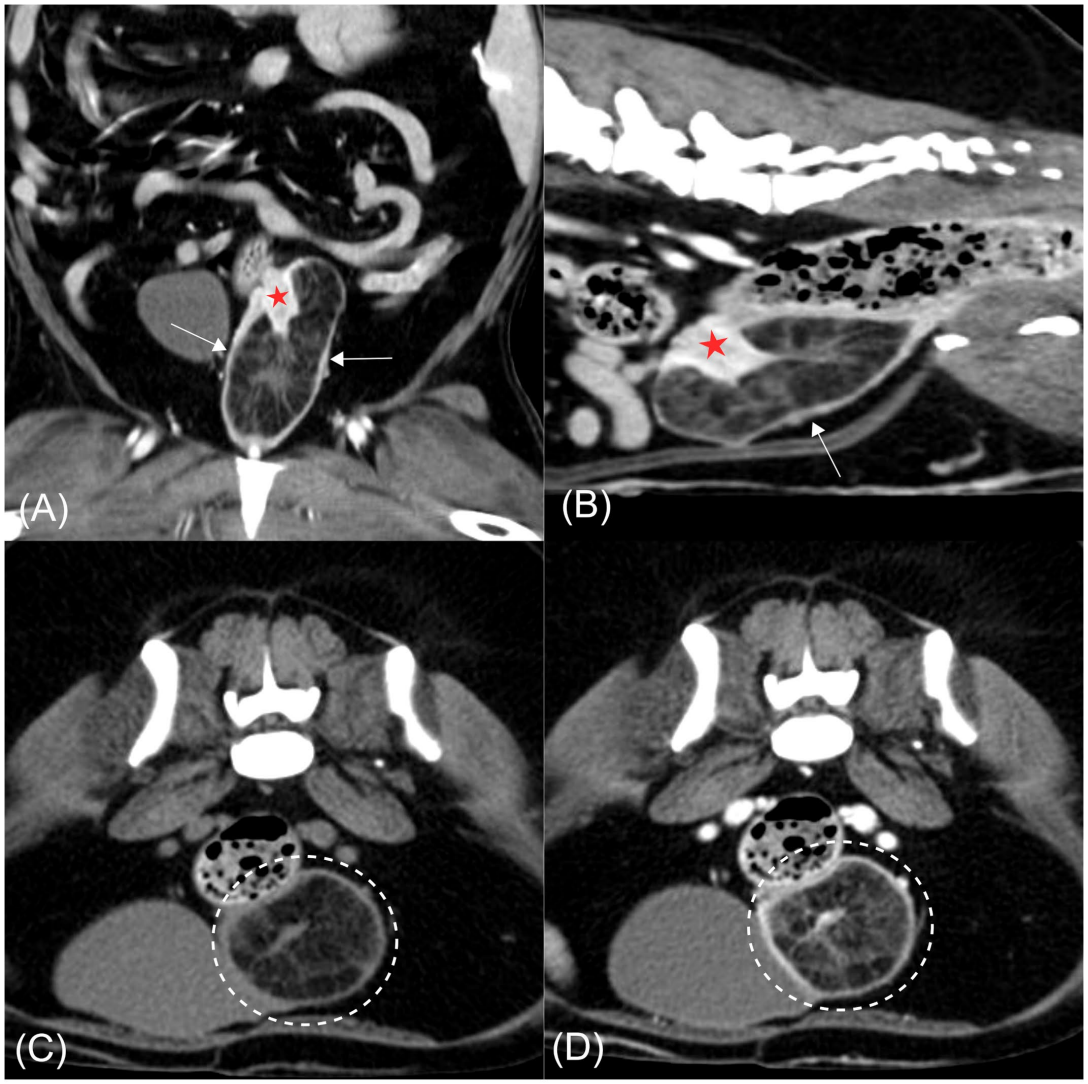
Computed tomography images of a hyperplastic cervical polyp with lipomatous differentiation in a dog. **(A)** Post-contrast coronal image. **(B)** Post-contrast sagittal image. **(C)** Pre-contrast transverse image. **(D)** Post-contrast transverse image. A well-defined hypoattenuating mass is evident at the level of the cervix, partially extending into the cranial vagina (arrow). The mass contains intraluminal soft tissue-attenuating striations continuous with the partially invaginated and contrast-enhancing cervical wall (red asterisk). In both transverse images **(C,D)**, hypoattenuating regions within the mass are consistent with fat attenuation and remain non-enhancing following contrast administration (dashed circle).

CT imaging revealed a well-defined hypoattenuating mass measuring approximately 37 × 35 × 73 mm, containing intraluminal soft tissue–attenuating striations, anatomically located at the level of the cervix and extending partially into the cranial vagina. The cervix appeared partially invaginated into the hypodense mass, with internal striations continuous with cervical tissue (Figures 2A,B). The invaginated cervical wall exhibited mild thickening and marked contrast enhancement relative to the vagina, aiding its radiological identification (19). Previous studies have reported that the cervix demonstrates a higher degree of contrast enhancement, reflected by higher attenuation values, than the vagina (19). In the present case, the conspicuous enhancement of the invaginated cervical wall was consistent with these findings (cervix, mean 183 HU; vagina, mean 160 HU), supporting the conclusion that the mass originated from the cervix rather than the vagina. Mild wall thickening and multiple small cystic lesions were also noted within the uterine horns and cervix. Both ovaries appeared unremarkable. The cervix showed mean attenuation values of 50 and 184 Hounsfield units (HU) on pre- and post-contrast CT images, respectively, consistent with soft tissue attenuation. The striated tissue contiguous with the cervix demonstrated HU values of 23 pre-contrast and 63 post-contrast, which fall within the range of soft tissue attenuation. In contrast, the hypoattenuating areas within the mass exhibited HU values of −113 pre-contrast and −90 post-contrast, consistent with fat attenuation (Figures 2C,D) (20).

To alleviate the clinical signs and facilitate mass removal, an ovariohysterectomy was performed. The preoperative medications included cefazolin (30 mg/kg; Cefazoline Inj. 1 g; Chong Kun Dang Pharmaceutical Corp., Seoul, Korea), fentanyl (0.003 mg/kg; Fentanyl Citrate Inj. 100 μg/2 mL; BC World Pharm Co., Ltd., Gyeonggi-do, Korea), famotidine (0.5 mg/kg; Gaster Inj. 20 mg/10 mL; Dong-A ST Co., Ltd., Seoul, Korea), and midazolam (0.1 mg/kg; Midacom Inj. 1 mg/1 mL; Myungmoon Pharm Co., Ltd., Seoul, Korea). Anesthesia was induced using intravenous propofol (4 mg/kg) and maintained using inhaled isoflurane (2.0–3.5%; Isosol Inhalation Solution; Kyungbo Pharm Co., Ltd., Seoul, Korea).

**Figure 3:**
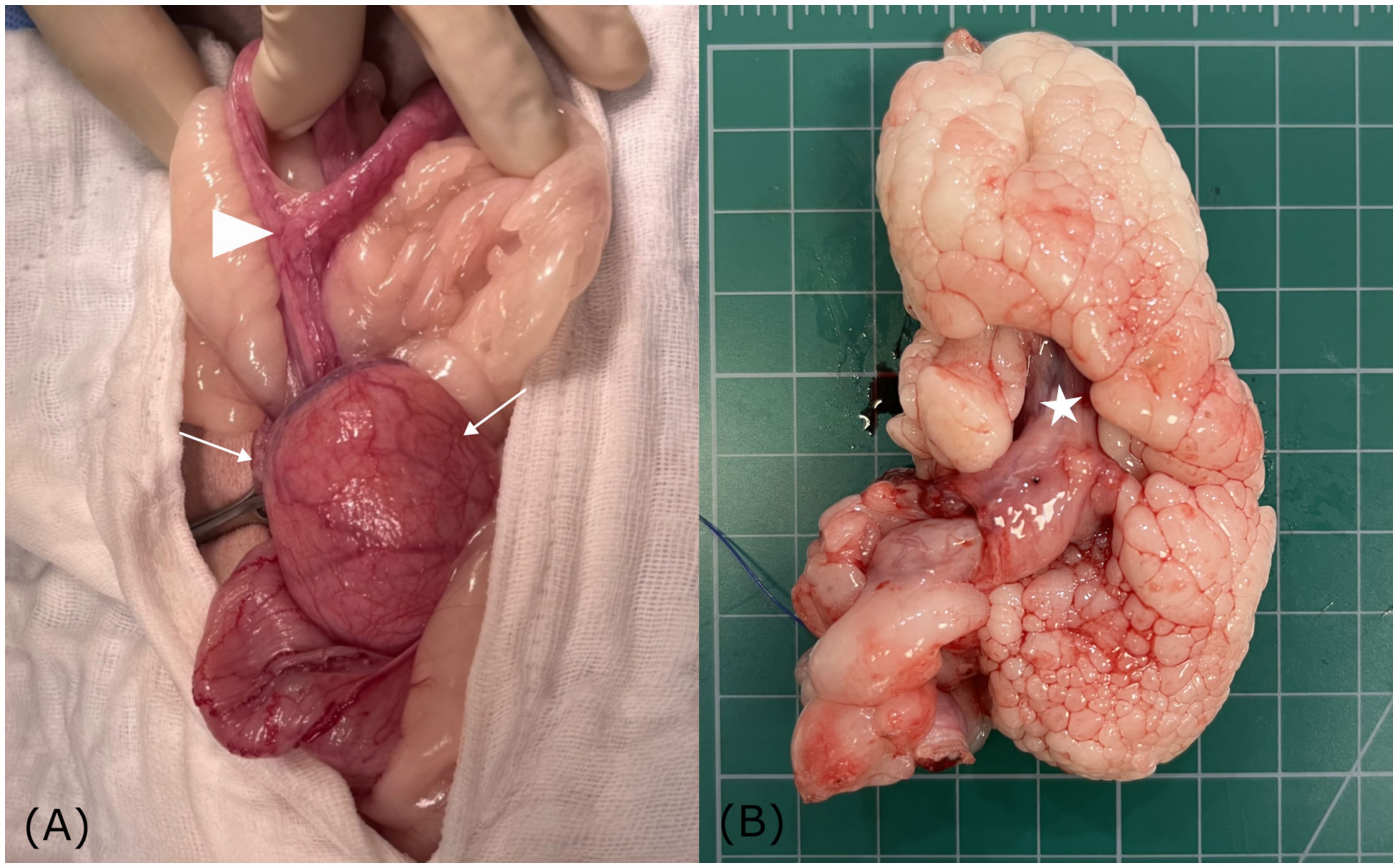
Gross anatomical findings of a hyperplastic cervical polyp with lipomatous differentiation in a dog. **(A)** Gross images showing both uterine horns converging at the uterine body (arrowhead), with the cervix embedded within a rounded mass (white arrow). **(B)** Sectioning showing a well-demarcated, multilobulated, polypoid mass, continuous with the inverted cervix (asterisk). The cauliflower-like appearance of the pale, soft to firm lobules suggests lipomatous differentiation.

Following abdominal incision, the left ureter was identified along the lateroventral aspect of the uterine mass and carefully isolated to prevent iatrogenic injury. The uterine body where both horns converged was visualized, with the cervix embedded within the rounded uterine mass (Figure 3A). Both ovaries and uterine horns were removed using a bipolar electrosurgical vessel-sealing device, which allowed secure hemostasis of the ovarian pedicles and broad ligaments. The cervix, incorporated within the mass, was isolated at its caudal margin, and the lower portion was ligated with absorbable sutures. The mass with the cervix was then excised using the same device. Estimated intraoperative blood loss was minimal, and routine three-layer closure was performed. The surgery was completed without complications. The patient recovered uneventfully from anesthesia.

The patient remained hospitalized for 3 days for postoperative monitoring, analgesia, supportive care, and wound management. Analgesia was initially provided with a continuous rate infusion (CRI) of remifentanil (Remiva Inj. 1 mg; Hana Pharm Co., Ltd., Hwaseong, Korea) administered intravenously for 15 h, followed by maintenance intravenous fluid therapy (Half-Sol Inj.; Dai Han Pharm Industrial Co., Ltd., Seoul, Korea) at a 1-fold rate for the remainder of the hospitalization. The surgical site was covered with a protective bandage, and the wound was disinfected twice daily with 0.2% chlorhexidine solution (Greenhexidine Sol. 5%; Green Pharmaceutical Co., Ltd., Seoul, Korea). At discharge, the patient was prescribed a three-day course of oral antibiotics, a gastroprotectant, and an anti-anaerobic agent. Detailed information on the administered drugs and wound management is provided in Supplementary Tables 1,2.

At the 2-week postoperative clinical check, the dog was in good health, with no recurrence of vaginal discharge or other abnormal signs observed. In addition, at a 3-month follow-up conducted by telephone consultation, the owner reported that the dog remained clinically normal without any recurrent symptoms. Because no postoperative complications or abnormal clinical signs were noted, no additional diagnostic examinations were performed.

**Figure 4:**
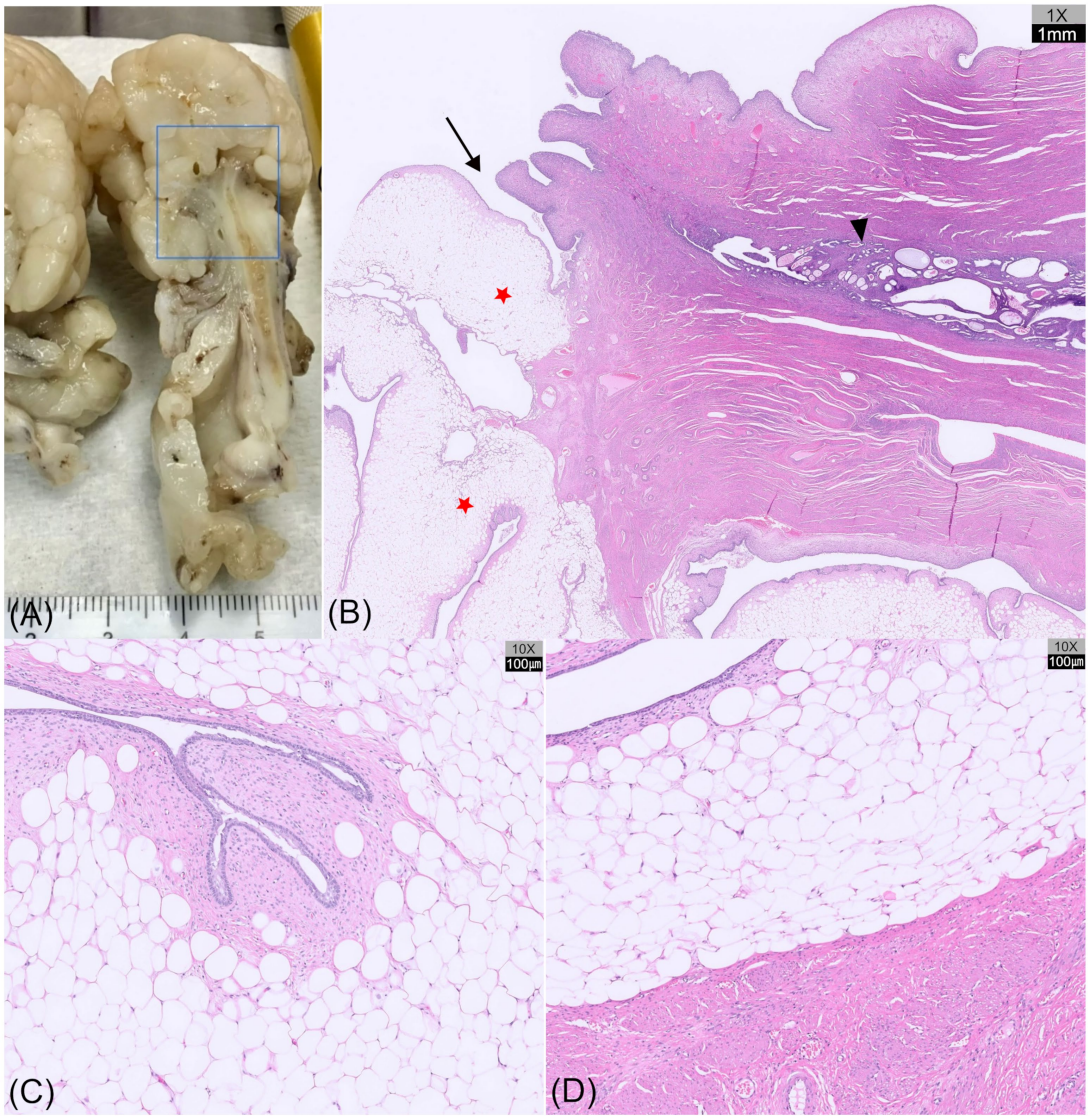
Gross sectioning and histopathological images of a hyperplastic cervical polyp with lipomatous differentiation in a dog (hematoxylin and eosin [HE] staining). **(A)** Tissue bisection showing a segment of the uterine body, followed by cervical tissue from which the mass appears to originate. **(B)** Histological section (H&E, ×1) of the region outlined by the blue box in **(A)** showing the uterine body with cystic endometrial glands (arrowhead), transitioning into cervical tissue (arrow), from which a multilobular, polypoid mass (red asterisks) originates. **(C)** Microscopic view (H&E, ×10) of the mass showing well-differentiated adipose tissue, with peripheral spindle cells and epithelial lining. The adipocytes are large, polygonal cells with a single lipid vacuole. **(D)** Abrupt transition between lipomatous tissue and the smooth muscle wall of the cervix (H&E, ×10).

On gross examination, sectioning of the excised uterine mass revealed an intraluminal, polypoid mass adhering to the cervical wall. The cervical mass was well-demarcated and multilobulated, exhibiting a nodular, cauliflower-like architecture. The coalescing lobules were pale and soft to firm in consistency, suggestive of a lipomatous component (Figures 3B and 4A). The uterine horn was moderately distended with wall thickening. Sectioning revealed an irregular endometrium containing multiple small white-to-translucent cystic structures projecting into the lumen. The uterus and associated masses were subjected to histopathological evaluation.

Histological examination revealed a segment of uterine body containing cystically dilated endometrial glands that transitioned into cervical tissue, from which the mass appeared to arise (Figure 4B). The polypoid mass was lined by columnar to transitional epithelium with variable numbers of goblet cells, and showed focal abrupt transitions to stratified squamous epithelium. These epithelial features are consistent with the normal histological characteristics of the canine cervix (21), thereby confirming a cervical rather than a vaginal origin of the mass. Microscopically, the mass exhibited multilobular polypoid proliferation composed predominantly of well-differentiated adipose tissue. The adipocytes were large polygonal cells with discrete borders, each containing a single large lipid vacuole that displaced the nucleus to the periphery (Figure 4C). Occasional clusters of foamy macrophages were scattered within sheets of adipose tissue. At the periphery, the lobules were bordered by moderately cellular spindle cells within the collagenous stroma and lined with 1–2 layers of epithelial cells, including areas with ciliated columnar cells along the mucosal surface (Figure 4D). Focal mucosal invaginations and scattered glandular structures were also observed.

Based on its anatomical location and histological features, the mass was diagnosed as a hyperplastic polyp with prominent lipomatous differentiation originating from the cervix.

## 3 Discussion

This case report describes a hyperplastic polyp with lipomatous differentiation originating from the cervix of a dog, a finding not previously documented in veterinary literature. Unlike previously reported uterine or vaginal polyps, this lesion was unique due to its cervical origin and its predominant composition of mature adipose tissue with polypoid proliferation.

The pathogenesis of cervical polyps in dogs remains poorly understood; however, mechanisms proposed in human medicine, such as estrogen-induced mucosal proliferation, hormonal imbalance, inflammation, and tissue regeneration, may be relevant (10, 22, 38). In the present case, the patient was an intact female dog with concurrent cystic endometrial hyperplasia (CEH), indicating repeated exposure to endogenous estrogen and progesterone (23). These hormonal influences may have contributed to hyperplastic changes not only in the endometrium but also in the cervical mucosa. Similar to humans, hormonally mediated mucosal responses likely play a key role in cervical polyp development.

Histologically, the polyp was composed predominantly of well-differentiated adipose tissue. Lipomatous masses of the female reproductive tract are rare in both humans and animals. In humans, these include pure lipomas; mixed lipomatous tumors such as lipoleiomyomas, angiomyolipomas, fibromyolipomas, and lipofibromas; and malignant neoplasms such as liposarcomas (24, 25). Similar lesions have been infrequently reported in dogs, including lipoleiomyomas of the vulva and uterus, angiolipoleiomyomas, lipomas, and liposarcomas (13–18). The lesion in this case should be differentiated from the aforementioned reproductive tract masses containing adipose tissue. Malignant neoplasms such as liposarcomas were excluded in the present case based on the absence of histological features typically associated with malignancy, including lipoblasts, cellular pleomorphism, and mitotic activity (26). Lipoleiomyomas are characterized by an admixture of proliferating smooth muscle and mature adipose tissues (25). However, the present case showed no definitive evidence of smooth muscle proliferation within the mass, making that diagnosis unlikely. While pure lipomas tend to be well-circumscribed and confined to the uterine wall or lumen, the multilobular, mucosa-lined structure of this lesion was more consistent with a mucosal polyp exhibiting lipomatous differentiation than a conventional lipoma (27).

The histogenesis of uterine lesions containing adipose tissue remains incompletely understood. In human medicine, the proposed mechanisms include adipocytic differentiation of mesenchymal progenitor cells, lipomatous metaplasia, and stem cell recruitment to the uterine stroma (24). In veterinary medicine, reports of fatty uterine lesions are extremely rare, and their pathogenesis remains largely unexplored. However, in the case of lipomas, which are among the most commonly diagnosed benign soft tissue tumors in dogs, the recognized risk factors include breed predisposition, advanced age, and obesity (28). In humans, lipomas are associated with genetic susceptibility, prior trauma, and metabolic disorders such as obesity, hyperlipidemia, and diabetes (29). The patient in the present case was markedly obese (body condition score 10/10) and demonstrated hypertriglyceridemia, suggesting that metabolic dysregulation may have contributed to lipomatous differentiation of the cervical polyp.

In dogs, reproductive tract diseases are typically diagnosed based on clinical signs and initial imaging modalities such as radiography and ultrasonography. Clinical signs associated with reproductive tract polyps are variable and may include vaginal discharge, lethargy, or abdominal enlargement, as reported in previous cases (1). In the present case, abnormal vaginal discharge was the primary complaint that prompted further diagnostic evaluation. Because such clinical signs are nonspecific, advanced imaging is often required to further delineate lesion characteristics. When neoplasia is suspected, particularly in cases with pelvic lesions that are difficult to evaluate with ultrasound alone, CT is often required to assess the lesion’s origin, composition, and potential metastasis (30). In the present case, a combination of radiography, ultrasonography, and CT was used to characterize the mass, which was notable for its distinct fat attenuation. In intact female dogs, the most commonly encountered reproductive tract disease is CEH–pyometra complex, which often presents with luminal fluid accumulation, causing the uterus to appear on radiographs as a soft-tissue density tubular structure in the region of the uterine horns and body. Although they are less common, uterine neoplasms may have similar radiographic characteristics (31). However, in the present case, the uterine lesion exhibited a distinctly radiolucent appearance consistent with fat attenuation, unlike the soft tissue opacity typically observed in common reproductive tract diseases. This radiolucency may represent a key imaging feature suggesting adipose content.

The ultrasonographic appearance of the lesion in this case differed from those of previously reported uterine malignancies and endometrial polyps in dogs. While such lesions can appear as solid masses, they often exhibit mixed cystic features associated with hemorrhage or necrosis (1, 30). In contrast, the lesion described here appeared as a well-defined solid mass lacking cystic components and displaying a predominantly homogeneous hyperechoic pattern. This echogenicity was suggestive of intralesional adipose tissue, which is an uncommon finding in canine uterine pathology.

Uterine lesions containing adipose tissue are extremely rare in dogs, with only two reports describing their imaging features in detail. One case involved a German Shepherd with a uterine liposarcoma that appeared as a mixed soft tissue opacity on radiographs and a heterogeneous hypoechoic mass with multiple cystic areas on ultrasonography (18). Another case described concurrent lipoleiomyoma and leiomyoma, in which the lipoleiomyoma exhibited fluid-filled cavities with soft tissue septations but lacked fat-specific attenuation on CT (14). In contrast, the uterine lesion in the present case demonstrated imaging features consistent with adipose tissue on radiography, ultrasonography, and CT. These findings closely resemble those reported in human uterine lipomas and lipoleiomyomas. Ultrasonography of lipoma and lipoleiomyomas revealed a well-defined, homogeneously hyperechoic intrauterine mass, a nonspecific finding in human cases (32–34). CT confirmed the cervical origin of the mass, which demonstrated fat attenuation with thin, contrast-enhancing internal striations, a pattern characteristic of human lipoleiomyomas. Unlike pure lipomas, which typically appear as uniformly fat-attenuating, non-enhancing masses encapsulated masses (35), lipoleiomyomas may show enhancing interlesional septa, a distinctive feature that was also identified in the present case. Because lipoleiomyomas vary in the proportion of adipose and smooth muscle tissues, the amount of soft tissue components differs by lesion, often resulting in a heterogeneous imaging appearance (36, 39). In the present case, the lesion consisted of lobules of well-differentiated adipocytes enclosed by mucosal epithelium, with an enhanced mucosal lining corresponding to striated soft tissue attenuation on CT. This appearance closely resembles the interlesional septa commonly observed in lipoleiomyomas. Therefore, in cases showing fat-attenuating uterine masses with enhancing septa on CT, the differential diagnosis should include not only lipoleiomyomas and other mixed lipomas but also multilobulated fatty-differentiated uterine polyps.

In human medicine, where surgical interventions are not immediately pursued, MRI is often used to improve diagnostic accuracy, with numerous reports supporting its utility. MRI offers superior soft tissue contrast compared to CT, allowing clearer delineation of the uterine wall and adjacent structures. It also enhances tissue characterization through fat signal loss on fat-suppressed T1- and T2-weighted sequences, facilitating the identification of intralesional fat (36). In veterinary practice, when surgery is not feasible owing to the patient’s overall condition or owner preference, MRI can aid in accurate differential diagnosis and treatment planning. Although MRI was not performed in the present case, fat-suppressed MRI sequences should be considered in similar cases to better characterize lesion origin and composition.

Although this lesion was histologically benign, reproductive tract masses in dogs may also include malignant neoplasms with overlapping imaging features, making accurate differentiation critical for treatment planning (30, 31, 37). While surgical excision is curative for benign polyps, suspected malignant lesions such as leiomyosarcoma may require wider resection and long-term oncologic monitoring (18). Intraoperative risks include hemorrhage or injury to adjacent urinary structures, though none occurred in this case. Postoperative care typically involves analgesia, antimicrobial therapy, and wound management, followed by clinical rechecks to ensure recovery. Although the present patient recovered uneventfully, extended surveillance is recommended given the rare possibility of recurrence or malignant transformation.

In conclusion, this is the first reported case of a hyperplastic polyp with adipose differentiation originating from the cervix in a dog. The lesion showed distinct anatomical and histopathological features, along with imaging characteristics, particularly fat-specific findings, on CT and ultrasonography, which differed from typical uterine conditions. These findings suggest that hormonal influences, a pathogenic mechanism proposed in human medicine, may also be relevant in veterinary cases. This report contributes to the limited literature on lipomatous uterine lesions in dogs and may serve as a useful reference for the diagnosis and differential assessment of similar cases in the future.

## Data Availability

The original contributions presented in the study are included in the article/Supplementary material, further inquiries can be directed to the corresponding author.
