# Supplementary material for: Case Report: Hyperplastic cervical polyp with lipomatous differentiation in a dog
Source: Front Vet Sci. 2025 Oct 22;12:1658919. doi: 10.3389/fvets.2025.1658919 (PMC12587459; doi:10.3389/fvets.2025.1658919)
Supplement: Supplementary file 1 [file Table_1.docx]

**Supplementary Table 1.**

**Drugs and wound management during hospitalization**

| **Treatment** | **Details** | **Frequency / Route** | **Product (manufacturer, country)** | **Purpose** |
| --- | --- | --- | --- | --- |
| Sulbactam/Ampicillin | 20 mg/kg | BID, IV | Ubacillin Inj. 750 mg (Kukje Pharm Co., Ltd., Korea) | Perioperative antimicrobial prophylaxis |
| Famotidine | 0.5 mg/kg | BID, IV | Gaster Inj. 20 mg/10 mL (Dong-A ST Co., Ltd., Korea) | Gastric acid suppression |
| Marbofloxacin | 2 mg/kg | SID, SC | Marbocyl® 2% Inj. (Vetoquinol S.A., France) | Broad-spectrum antibiotic |
| Metronidazole | 10 mg/kg | BID, IV | Meckool Inj. 5 mg/2 mL (Jeil Pharm Co., Ltd., Korea) | Anaerobic bacterial coverage |
| Wound management | Bandaging + disinfection with 0.2% chlorhexidine | Bandage: continuous / disinfection: BID | Greenhexidine Sol. 5% (Green Pharmaceutical Co., Ltd., Korea) | Local wound care, infection control |

**Supplementary Table 2.**

**Discharge medications (3 days, PO)**

| **Drug (generic)** | **Dose** | **Frequency / Route** | **Product (manufacturer, country)** | **Purpose** |
| --- | --- | --- | --- | --- |
| Ampicillin | 20 mg/kg | BID, PO | Ampicillin Cap. 500 mg (Chong Kun Dang Pharm, Korea) | Antimicrobial |
| Famotidine | 0.5 mg/kg | BID, PO | Famotidine Tab. 20 mg (Nelson Pharm, Korea) | Gastric acid suppression |
| Marbofloxacin | 2.75 mg/kg | SID, PO | Marbocyl® P 20 mg Tab. (Vetoquinol S.A., France) | Broad-spectrum antibiotic |
| Metronidazole | 15 mg/kg | BID, PO | Flasinyl® Tab. 250 mg (HK inno.N Corp., Korea) | Anaerobic bacterial coverage |
